# Aiding Large Language Models Using Clinical Scoresheets for Neurobehavioral Diagnostic Classification From Text: Algorithm Development and Validation

**DOI:** 10.2196/75030

**Published:** 2025-10-21

**Authors:** Kaiying Lin, Abdur Rasool, Saimourya Surabhi, Cezmi Mutlu, Haopeng Zhang, Dennis P Wall, Peter Washington

**Affiliations:** 1 Institute of Linguistics Academia Sinica Taipei Taiwan; 2 University of Hawaiʻi at Mānoa Honolulu, HI United States; 3 Stanford University Stanford, CA United States; 4 University of California, San Francisco San Francisco United States

**Keywords:** neurological diagnostics, classification, large language model, LLM, chatbot, artificial intelligence, AI

## Abstract

**Background:**

Large language models (LLMs) have demonstrated the ability to perform complex tasks traditionally requiring human intelligence. However, their use in automated diagnostics for psychiatry and behavioral sciences remains under-studied.

**Objective:**

This study aimed to evaluate whether incorporating structured clinical assessment scales improved the diagnostic performance of LLM-based chatbots for neuropsychiatric conditions (we evaluated autism spectrum disorder, aphasia, and depression datasets) across two prompting strategies: (1) direct diagnosis and (2) code generation. We aimed to contextualize LLM-based diagnostic performance by benchmarking it against prior work that applied traditional machine learning classifiers to the same datasets, allowing us to assess whether LLMs offer competitive or complementary capabilities in clinical classification tasks.

**Methods:**

We tested two approaches using ChatGPT, Gemini, and Claude models: (1) direct diagnostic querying and (2) execution of chatbot-generated code for classification. Three diagnostic datasets were used: ASDBank (autism spectrum disorder), AphasiaBank (aphasia), and Distress Analysis Interview Corpus-Wizard-of-Oz interviews (depression and related conditions). Each approach was evaluated with and without the aid of clinical assessment scales. Performance was compared to existing machine learning benchmarks on these datasets.

**Results:**

Across all 3 datasets, incorporating clinical assessment scales led to little improvement in performance, and results remained inconsistent and generally below those reported in previous studies. On the AphasiaBank dataset, the direct diagnosis approach using ChatGPT with GPT-4 produced a low *F*_1_-score of 65.6% and specificity of 33%. The code generation method improved results, with ChatGPT with GPT-4o reaching an *F*_1_-score of 81.4%, specificity of 78.6%, and sensitivity of 84.3%. ChatGPT with GPT-o3 and Gemini 2.5 Pro performed even better, with *F*_1_-scores of 86.5% and 84.3%, respectively. For the ASDBank dataset, direct diagnosis results were lower, with *F*_1_-scores of 56% for ChatGPT with GPT-4 and 54% for ChatGPT with GPT-4o. Under code generation, ChatGPT with GPT-o3 reached 67.9%, and Claude 3.5 performed reasonably well with 60%. Gemini 2.5 Pro failed to respond under this assessment condition. In the Distress Analysis Interview Corpus-Wizard-of-Oz dataset, direct diagnosis yielded high accuracy (70.9%) but poor *F*_1_-scores of 8% using ChatGPT with GPT-4o. Code generation improved specificity—88.6% with ChatGPT with GPT-4o—but *F*_1_-scores remained low overall. These findings suggest that, while clinical scales may help structure outputs, prompting alone remains insufficient for consistent diagnostic accuracy.

**Conclusions:**

Current LLM-based chatbots, when prompted naively, underperform on psychiatric and behavioral diagnostic tasks compared to specialized machine learning models. Clinical assessment scales might modestly aid chatbot performance, but more sophisticated prompt engineering and domain integration are likely required to reach clinically actionable standards.

## Introduction

### Background

Large language models (LLMs) have recently demonstrated capabilities that closely approximate or exceed human cognitive functions in various domains [1-3]. Given their efficacy in executing complex tasks, there is a burgeoning interest in exploring the potential applications of LLMs in clinical settings, including in areas such as providing emotional support [4,5] and mental health diagnoses [6-10]. The diagnostic process for neurobehavioral conditions typically encompasses comprehensive clinical assessments and longitudinal behavioral observations [11,12]. The integration of LLMs (as well as other machine learning [ML] models) into this process could potentially streamline this complex and time-consuming diagnostic procedure by facilitating automated screening processes [13,14]. While some research highlights some of the potential challenges of using LLMs for these tasks [15-18], they are emerging as a promising avenue for developing scalable and accessible screening services.

ChatGPT [19], Gemini [20], and Claude [21], prominent LLM-based conversational agents, have been the subject of evaluation for their potential in digital neurobehavioral diagnostics. Previous studies have indicated that the capabilities of chatbots for neurobehavioral classification remain limited even when assessing specific conditions or smaller patient cohorts [10,22,23]. In response to these findings, subsequent research efforts have focused on enhancing ChatGPT’s performance in this domain [6,22], typically using varied prompting strategies such as the formulation of precise inquiries and the provision of relevant contextual information.

### Objectives

Building on these foundational works, we aimed to evaluate the use of LLM-based chatbots to aid in automated diagnostics for neuropsychiatric conditions using assessment scales. We evaluated two paradigms: (1) directly deriving diagnoses from textual data and (2) chatbot-generated code executed in a local environment for diagnostic classification. As an attempt to reach clinical relevance, we instructed the chatbots to either provide ratings on standardized clinical assessment scales which were then used to derive a final diagnosis or to incorporate these ratings into their diagnostic decision making. This approach aimed to leverage established clinical approaches to diagnosis while harnessing the analytical capabilities of LLMs. We hypothesized that offering this clinically grounded method for automated diagnosis would lead to improved diagnostic performance.

## Methods

### Overview

**Figure 1:**
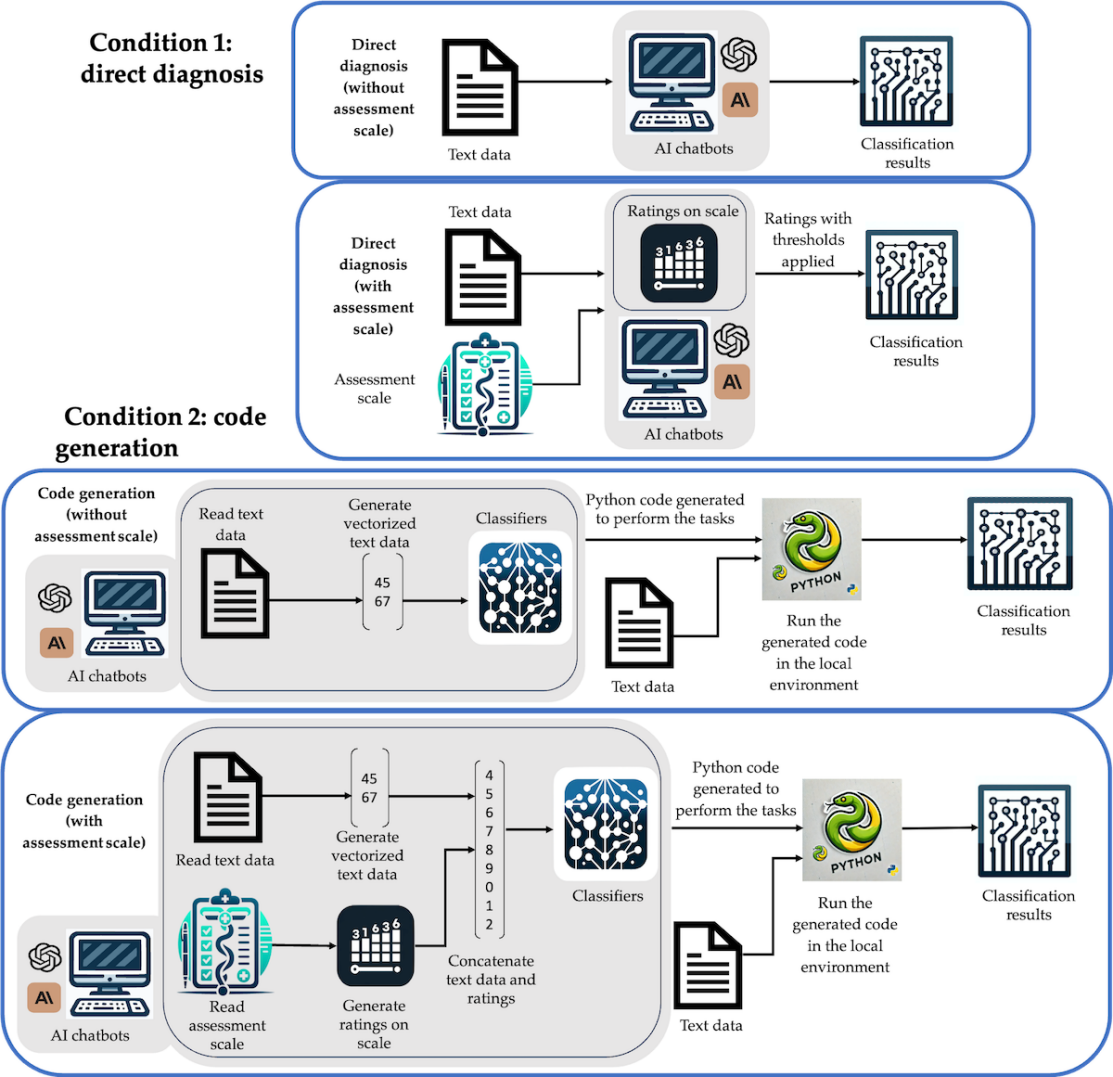
The 4 methods we explored in this study. The top 2 panels illustrate the direct diagnosis approach with and without the use of assessment scales, which require the chatbots to directly provide predictions or ratings on assessment scales. The bottom 2 panels, consisting of the code generation approach with and without assessment scales, require the chatbots to generate code that is subsequently executed in a Python environment.

We implemented 4 distinct methodologies to evaluate the diagnostic capabilities of the chatbots (Figure 1). In the direct diagnosis approach without assessment scales, we directly input data into the conversational artificial intelligence (AI) model, which then generated classification results. This process involved providing the chatbot with the processed dataset as input data and defining its primary task as providing neurobehavioral classification results for the condition of interest. We instructed the chatbot to derive diagnostic classifications for all participants using the text data from the entire processed dataset. If the chatbot indicated an inability to perform this task and requested to run in our environment, we directed it to use its pretrained knowledge to complete the task (see Multimedia Appendix 1 for the detailed prompts of each condition).

The direct diagnosis approach using assessment scales involved inputting both data and a clinical assessment scale into the model; the model was subsequently tasked with rating items on the scale and providing these ratings as output. We then applied predefined thresholds from the clinical assessment scales to each data subject’s ratings to derive final neurobehavioral diagnoses.

For both direct diagnosis conditions, we performed zero-shot classification without conducting training and testing splits, as we aimed to evaluate the models’ ability to generalize from their pretrained knowledge. This process was repeated 5 times for each condition, with results averaged across iterations to improve robustness.

In addition to direct diagnosis, we explored a code generation approach to determine whether LLM-based chatbots could perform automated neurobehavioral classification by externalizing their reasoning into executable models. The motivation for the code generation condition stemmed from the observation that LLMs such as ChatGPT often struggle with directly solving complex reasoning tasks (at the time of our experiments) but can excel at generating code that reliably solves these problems. A notable example is that while ChatGPT frequently fails to correctly solve math puzzles through direct reasoning, it can generate Python code that solves them efficiently when executed. Recent studies have examined this phenomenon, revealing that LLMs perform poorly on complex logic-based tasks when relying solely on their internal reasoning capabilities yet demonstrate improved performance when prompted to generate code that encodes the required logic [24,25]. This suggests that the reasoning and structure embedded in generated code may enable LLMs to circumvent some of the limitations they face during direct response generation. While the diagnostic processes under the code generation condition differed fundamentally from those under the direct diagnosis condition, they offer a complementary perspective on the models’ capabilities. Notably, the chatbots frequently produced executable code—even without explicit prompting—in our early pilot tests.

In the code generation approach without assessment scales, which served as a control condition, we fed the processed data as input into the conversational AI model. Following the data review, we instructed the chatbot to select what it deemed the most appropriate algorithm for the task and output the corresponding Python code. This code was subsequently executed in an external Python environment (Python Software Foundation). We tasked the chatbot with conducting stratified 5-fold cross-validation on the dataset, reporting *F*_1_-score, specificity, sensitivity, and accuracy as performance metrics. To optimize results, we engaged in an iterative process with the chatbot, requesting performance improvements until the generated code produced results consistent with its previous 2 iterations.

The code generation approach using assessment scales began with providing the chatbot with the processed data as input followed by a standardized assessment scale. We then prompted the model to generate the code and apply established cutoff thresholds from these scales as output to determine the final diagnosis. However, observing that this often resulted in unsatisfactory classifications, we next encouraged the chatbots to incorporate these ratings into an ML algorithm of their own design. The chatbots produced the algorithm, which we then ran in our local environment. If no further performance improvements from the condition without assessment scales were observed, we directed the chatbot to revert to the algorithm used in the condition without the assessment scale and integrate the assessment scale ratings into the training procedures. This methodology facilitated a direct comparison of performance between 2 code generation approaches, making any potential improvements attributable to the assessment scale approach. (see Multimedia Appendix 2 for an example of generated code).

Table 1 shows the final algorithm used in each code generation condition. We ensured that the chatbots incorporated the assessment scale ratings into their algorithms, requesting integration if they were initially omitted. The iterative process for each condition continued until the performance of the generated code reached a plateau with no significant improvement observed over 2 consecutive iterations.

**Table 1:** Machine learning algorithms produced by the chatbots in the 2 code generation conditions.

Approach			Algorithms				
			AphasiaBank		ASDBank		DAIC-WOZ^a^ database
**Code generation**―**no assessment scale**							
	GPT-4	TF-IDF^b^+LR^c^		TF-IDF+LR		CountVectorizer+LR	
	GPT-4o	Word Count		CountVectorizer+LR		TF-IDF+XGB^d^ classifier	
	Claude 3.5	Word Count		TF-IDF+LR		TF-IDF+LR	
	GPT-o3	TF-IDF+LR		TF-IDF+LR		TF-IDF+LinearSVC	
	Gemini 2.5 Pro	TF-IDF+LR		TF-IDF+LinearSVC		Sentence embedding+LinearSVC	
**Code generation**―**assessment scale**							
	GPT-4	TF-IDF+LR		TF-IDF+LR		CountVectorizer+LR	
	GPT-4o	Word Count+LR		CountVectorizer+LR		TF-IDF+XGB classifier	
	Claude 3.5	Word Count+threshold		TF-IDF+RF^e^		TF-IDF+LR	
	GPT-o3	TF-IDF+LR		TF-IDF+LR		TF-IDF+LinearSVC	
	Gemini 2.5 Pro	TF-IDF+LR		—^f^		Sentence embedding+LinearSVC	

^a^DAIC-WOZ: Distress Analysis Interview Corpus-Wizard-of-Oz.

^b^TF-IDF: term frequency–inverse document frequency.

^c^LR: logistic regression.

^d^XGB: extreme gradient boosting.

^e^RF: random forest.

^f^Not applicable.

To assess statistical significance, we conducted 1000-fold permutation tests on the *F*_1_-score and accuracy to compare (1) direct diagnosis and code generation in non–assessment scale setups and (2) non–assessment scale and assessment scale setups. For comparisons between direct diagnosis and code generation, we aligned predictions by matching test sets across folds, comparing the performance on the same data points in both conditions and averaging the results across folds. For the checklist comparison, we ran permutation tests over all predictions from the respective conditions, also averaging across folds. Comparisons were omitted in cases in which the prediction patterns were apparently random or when performance in the latter condition was lower than in the former (ie, assessment scale<non–assessment scale or code generation<direct diagnosis).

### Datasets

We used 3 distinct databases, each focusing on a specific neurobehavioral condition. Two of these, ASDBank [26] and AphasiaBank [27], are sourced from TalkBank [28] and contain language samples for autism spectrum disorder (ASD) and aphasia, respectively, whereas the third database, the Distress Analysis Interview Corpus-Wizard-of-Oz (DAIC-WOZ) database [29], contains textual data from patients with depression, anxiety, and posttraumatic stress disorder.

AphasiaBank [27] is a repository containing multimedia language samples from both participants with aphasia and control participants. These samples were collected through standardized discourse tasks, including unstructured speech samples, picture descriptions, story narratives, and procedural discourse.

ASDBank [26] comprises a collection of language samples and interactions from individuals diagnosed with ASD. The data within ASDBank include transcribed audio and video recordings of clinical interviews and naturalistic interactions.

We used all available English-language transcripts from both AphasiaBank and ASDBank. Data processing was performed to consolidate all samples from a single participant into 1 data point. The resulting dataset comprised 715 aphasia data points and 352 control data points for AphasiaBank and 34 ASD data points and 44 control data points for ASDBank.

The DAIC-WOZ database [29] consists of semistructured interviews conducted by a simulated agent designed to identify symptoms of depression and posttraumatic stress disorder. These interviews include questions about personal experiences, quality of life, and emotions. We consolidated all samples from a participant, including the interviewer’s input, into a single data point. The DAIC-WOZ database includes 56 patient data points and 133 control data points.

Table 2 provides a summary of the diagnosis distribution across each dataset.

**Table 2:** The number of control and patient data points in each of the datasets we evaluated.

Database	Number of control data points	Number of data points for condition of interest
AphasiaBank	352	715
ASDBank	44	34
DAIC-WOZ^a^	133	56

^a^DAIC-WOZ: Distress Analysis Interview Corpus-Wizard-of-Oz.

Aphasia, depression, and ASD each manifest distinct linguistic characteristics that are both overlapping and unique. Aphasia, typically resulting from brain damage, is characterized by impaired language production and comprehension, often including repetitive language and the frequent use of filler words as individuals struggle to retrieve or organize words effectively [30]. Depression, while primarily a mood disorder, affects language through reduced verbal output, monotone speech, and a preference for negative or self-critical language patterns. Depressive language, such as expressions of negativity, can be a key symptom of the condition. Another characteristic linguistic feature is an excessive number of sighs, reflecting physical or emotional fatigue. ASD is marked by unique communication challenges, including delayed speech development, echolalia (repetition of phrases), difficulty with pragmatic language (eg, understanding sarcasm or social cues), and overly literal or formal speech. Individuals with ASD may also exhibit fragmented sentences and frequent use of filler words, reflecting challenges in organizing thoughts or navigating social interactions [31].

Many previous studies have leveraged the datasets we used in our research. However, much of the existing work has focused on advanced tasks such as multimodal detection or severity classification rather than simpler text-based binary classification using chatbots. These studies have often achieved strong (although not clinically translatable) performances, frequently exceeding 80% in *F*_1_-scores or accuracy. For example, Dinkel et al [32] applied a text-based multitask network to the DAIC-WOZ dataset, achieving an *F*_1_-score of 0.84 for binary detection. Similarly, Agrawal and Mishra [33] used a fused bidirectional encoder representation from transformers–a bidirectional long short-term memory model integrated with Extreme Gradient Boosting to perform binary classification, achieving an *F*_1_-score of 91%.

For the AphasiaBank dataset, most previous studies have focused on severity classification, making direct comparisons with our binary classification study challenging. The only relevant work, conducted by Cong et al [34], found that using LLM-derived surprisal features facilitated detection, achieving 79% in both accuracy and *F*_1_-score. Similarly, studies involving the ASDBank dataset are limited, partly due to its recent development. Chu et al [35] included another dataset, the Child Language Data Exchange System, as a source of healthy control data. By extracting a few linguistic features from these 2 datasets, their binary classification approaches reached an *F*_1_-scores of over 80% [35].

These studies suggest that LLM-based models directly diagnosing from the datasets used in this study should achieve high performance if chatbots exhibit comparable classification capabilities to those models in the previous studies.

### Models

We evaluated 2 approaches using 3 types of state-of-the-art conversational AI models: ChatGPT with GPT-4, ChatGPT with GPT-4o, and ChatGPT with GPT-o3 (OpenAI); Gemini 2.5 Pro (Google AI); and Claude 3.5 Sonnet (Anthropic). These models were selected because they are some of the most widely used modern LLMs and because their efficacy in neurobehavioral classification tasks remains underexamined in the current literature. Notably, models such as Gemini 2.5 Pro and ChatGPT with GPT-o3 incorporate built-in prompting strategies such as chain-of-thought reasoning, allowing us to examine how such strategies influence performance. We excluded open models such as Llama because they do not support file input and including them would require a different approach from that used for the other models we tested.

### Assessment Scales

We incorporated 3 widely recognized assessment scales and checklists used in clinical settings. We selected scales that assess behaviors at least tangentially related to language and that do not require extended observation periods. For example, the Autism Spectrum Quotient evaluates traits such as social preferences (“S/he prefers to do things with others rather than on her/his own”), behavioral patterns (“S/he prefers to do things the same way over and over again”), and attention capabilities (“have difficulty sustaining attention in tasks or fun activities”). The rating system for this checklist—*definitely disagree*, *slightly disagree*, *slightly agree*, and *definitely agree*—does not necessitate longitudinal observation, unlike scales that use time-sensitive ratings such as *rarely*, *less often*, *very often*, and *always*.

The assessment scales and checklists included in our study were as follows: (1) the fluency test in the Western Aphasia Battery–Aphasia Quotient (AphasiaBank) [36], (2) the Autism Spectrum Quotient (ASDBank) [37], and (3) Burn’s Depression Checklist [38] (DAIC-WOZ database).

In the 2 direct diagnosis conditions, we conducted the experimental procedure 5 times and obtained results based on the entirety of each dataset. We did not perform a training and testing split for these conditions, opting instead for a zero-shot classification approach to assess the models’ ability to generalize from their pretrained knowledge. However, in the code generation conditions, we instructed the chatbot to perform stratified 5-fold cross-validation on the entire dataset. The training and testing split ratio during each fold was 4:1. Results were evaluated based on the test sets generated during each fold and subsequently averaged.

### Ethical Considerations

This study did not involve the recruitment of human participants or the collection of new data. All analyses were conducted on publicly available, deidentified datasets**―**AphasiaBank, the DAIC-WOZ database, and ASDBank**―**that are widely used in research and do not contain personally identifiable information. As such, no application for ethics review was submitted. This approach is consistent with institutional and regional guidelines that exempt studies using publicly available, deidentified data from human subjects review.

## Results

### Core Results

Tables 3 to 8 present the cross-validation results of the 2 approaches applied to each dataset, reporting accuracy, *F*_1_-score, specificity, and sensitivity. Performance under the direct diagnosis conditions varied across datasets.

**Table 3:** Results of 4 approaches on the AphasiaBank dataset in the direct diagnosis condition.

			Accuracy	*F*_1_-score		Specificity		Sensitivity
Results from Cong et al [34]			0.79	0.79		―^a^		0.79
**No assessment scale, mean (SD)**								
	GPT-4	0.567 (0.1)		0.6556 (0.136)	0.33 (0.3)		0.684 (0.29)	
	GPT-4o	0.561 (0.029)		0.648 (0.111)	0.397 (0.11)		0.642 (0.22)	
	GPT-o3	0.49 (0.06)		0.544 (0.113)	0.328 (0.01)		0.665 (0.01)	
	Gemini 2.5 Pro	0.508 (0.01)		0.599 (0.012)	0.317 (0.02)		0.659 (0.013)	
**Assessment scale, mean (SD)**								
	GPT-4	0.293 (0.34)^b^		0.358 (0.376)^b^	0.297 (0.187)		0.647 (0.09)	
	GPT-4o	0.497 (0.01)^b^		0.55 (0.02)^b^	0.577 (0.02)		0.458 (0.02)	
	GPT-o3	0.555 (0.183)^c^		0.568 (0.4)^c^	0.108 (0.19)		0.645 (0.037)	
	Gemini 2.5 Pro	0.661 (0.07)^b^		0.792 (0.003)^b^	0.381 (0.08)		0.672 (0.003)	

^a^Missing data.

^b^No test conducted.

^c^*P*<.001 for GPT-o3 accuracy; *P<*.001 for *F*_1_-score (no assessment scale vs assessment scale).

**Table 4:** Results of 4 approaches on the AphasiaBank dataset in the code generation condition.

		Accuracy, mean (SD)	*F*_1_-score, mean (SD)	Specificity, mean (SD)	Sensitivity, mean (SD)
**No assessment scale**					
	GPT-4	0.67 (0.16)^a^	0.74 (0.17)^a^	0.79 (0.24)	0.40 (0.31)
	GPT-4o	0.67 (0.0113)^a^	0.802 (0.008)^a^	0.68 (0.011)	1 (0)
	GPT-o3	0.835 (0.035)^a^	0.865 (0.029)^a^	0.920 (0.077)	0.793 (0.041)
	Claude 3.5	0.605 (0.034)^b^	0.623 (0.036)^b^	0.844 (0.037)	0.488 (0.033)
	Gemini 2.5 Pro	0.7882 (0.02)^a^	0.8429 (0.016)^a^	0.6645 (0.057)	0.8490 (0.031)
**Assessment scale**					
	GPT-4	0.67 (0.16)^b^	0.74 (0.17)^b^	0.80 (0.25)	0.41 (0.30)
	GPT-4o	0.741 (0.022)^c^	0.814 (0.016)^c^	0.786 (0.024)	0.843 (0.007)
	GPT-o3	0.835 (0.035)^b^	0.865 (0.029)^b^	0.920 (0.077)	0.793 (0.041)
	Claude 3.5	0.608 (0.036)^b^	0.627 (0.039)^b^	0.844 (0.037)	0.492 (0.036)
	Gemini 2.5 Pro	0.7891 (0.021)^b^	0.8437 (0.015)^b^	0.6674 (0.072)	0.8490 (0.024)

^a^*P*<.001 for GPT-4 accuracy; *P*<.001 for GPT-4 *F*_1_-score; *P*<.001 for GPT-4o accuracy; *P*<.001 for GPT-4o *F*_1_-score; *P*<.001 for GPT-o3 accuracy; *P*<.001 for GPT-o3 *F*_1_-score; *P*<.001 for Gemini 2.5 Pro accuracy; *P<*.001 for Gemini 2.5 Pro *F*_1_-score (direct diagnosis versus code generation in non–assessment scale setups when marked in the “No assessment scale” section)

^b^No test conducted.

^c^*P*=.07 for GPT-4o accuracy; *P*=.06 for GPT-4o F1-score (assessment vs no assessment).

**Table 5:** Results of 4 approaches on the ASDBank dataset in the direct diagnosis condition.

			Accuracy	*F*_1_-score		Specificity		Sensitivity
Results from Chu et al [35]			0.76	0.85		0.2		0.94
**No assessment scale, mean (SD)**								
	GPT-4	0.5 (0.00)		0.598 (0.00)	0.227 (0.00)		0.853 (0.00)	
	GPT-4o	0.421 (0.03)		0.514 (0.129)	0.155 (0.212)		0.765 (0.323)	
	GPT-o3	0.6026 (0.00)		0.575 (0.00)	0.667 (0.00)		0.575 (0.00)	
	Gemini 2.5 Pro	0.485 (0.08)		0.449 (0.09)	0.549 (0.08)		0.421 (0.08)	
**Assessment scale, mean (SD)**								
	GPT-4	0.427 (0.01)^a^		0.56 (0.08)^a^	0.09 (0.157)		0.863 (0.24)	
	GPT-4o	0.491 (0.09)^a^		0.542 (0.117)^a^	0.236 (0.39)		0.802 (0.342)	
	GPT-o3	0.436 (0.00)^a^		0.607 (0.00)^a^	0.00 (0.00)		0.436 (0.00)	

^a^No test conducted.

**Table 6:** Results of 4 approaches on the ASDBank dataset in the code generation condition.

			Accuracy, mean (SD)	*F*_1_-score, mean (SD)		Specificity, mean (SD)		Sensitivity, mean (SD)
**No assessment scale**								
	GPT-4	0.618 (0.125)^a^		0.616 (0.104)^a^	0.55 (0.286)		0.71 (0.199)	
	GPT-4o	0.653 (0.103)^a^		0.55 (0.184)^a^	0.73 (0.303)		0.576 (0.378)	
	GPT-o3	0.679 (0.041)^a^		0.679 (0.041)^a^	0.864 (0.083)		0.433 (0.195)	
	Claude 3.5	0.68 (0.16)^b^		0.6 (0.22)^b^	0.67 (0.35)		0.69 (0.4)	
	Gemini 2.5 Pro	0.74 (0.09)^a^		0.63 (0.14)^a^	0.52 (0.16)		0.91 (0.08)	
**Assessment scale**								
	GPT-4	0.642 (0.165)^c^		0.628 (0.17)^c^	0.6 (0.334)		0.695 (0.231)	
	GPT-4o	0.628 (0.194)^b^		0.592 (0.1974)^b^	0.689 (0.325)		0.578 (0.257)	
	GPT-o3	0.679 (0.041)^b^		0.679 (0.041)^b^	0.864 (0.083)		0.433 (0.195)	
	Claude 3.5	0.64 (0.13)^b^		0.6 (0.23)^b^	0.69 (0.41)		0.67 (0.36)	

^a^*P=*.002 for GPT-4 accuracy; *P=*.001 for GPT-4 *F*_1_-score; *P=*.03 for GPT-4o accuracy; *P=*.015 for GPT-4o *F*_1_-score; *P=*.009 for GPT-o3 accuracy; *P=*.005 for GPT-o3 *F*_1_-score; *P=*.006 for Gemini 2.5 Pro accuracy; *P=*.003 for Gemini 2.5 Pro *F*_1_-score (direct diagnosis versus code generation in non–assessment scale setups when marked in the “No assessment scale” section)

^b^No test conducted.

^c^*P=*.99 and *P=*.99 for GPT-4 accuracy and F1-score (assessment vs non-assessment).

**Table 7:** Results of 4 approaches on the Distress Analysis Interview Corpus-Wizard-of-Oz (DAIC-WOZ) database in the direct diagnosis condition.

			Accuracy		*F*_1_-score		Specificity		Sensitivity
Results from Dinkel et al [32]			0.86		0.84		―		0.83
Results from Agrawal and Mishra [33]			―		0.91		―		0.89
**No assessment scale, mean (SD)**									
	GPT-4	0.333 (0.04)		0.452 (0.04)		0.08 (0.51)		0.939 (0.039)	
	GPT-4o	0.623 (0.01)		0.346 (0.176)		0.711 (0.168)		0.409 (0.347)	
	GPT-o3	0.595 (0.05)		0.252 (0.12)		0.704 (0.02)		0.269 (0.05)	
	Gemini 2.5 Pro	0.616 (0.11)		0.222 (0.132)		0.700 (0.02)		0.294 (0.09)	
**Assessment scale, mean (SD)**									
	GPT-4	0.56 (0.06)^a^		0.416 (0.07)^a^		0.56 (0.08)		0.516 (0.05)	
	GPT-4o	0.709 (0.05)^a^		0.08 (0.01)^a^		1 (0.00)		0.429 (0.006)	
	GPT-o3	0.635 (0.05)^b^		0.281 (0.14)^b^		0.72 (0.01)		0.355 (0.08)	
	Gemini 2.5 Pro	0.54 (0.07)^a^		0.363 (0.09)^a^		0.71 (0.06)		0.306 (0.08)	

^a^No test conducted.

^b^*P=*.44 for GPT-4 accuracy and *P=*.43 for *F*_1_-score (assessment vs no assessment).

**Table 8:** Results of 4 approaches on the Distress Analysis Interview Corpus-Wizard-of-Oz (DAIC-WOZ) database in the code generation condition.

		Accuracy, mean (SD)	*F*_1_-score, mean (SD)	Specificity, mean (SD)	Sensitivity, mean (SD)
**No assessment scale**					
	GPT-4	0.624 (0.024)^a^	0.268 (0.047)^a^	0.79 (0.035)	0.233 (0.048)
	GPT-4o	0.681 (0.126)^a^	0.2038 (0.1474)^a^	0.886 (0.087)	0.2286 (0.2382)
	GPT-o3	0.6667 (0.0572)^a^	0.1472 (0.1636)^a^	0.1091 (0.1185)	0.1091 (0.1185)
	Claude 3.5	0.649 (0.103)^b^	0.2386 (0.113)^b^	0.7672 (0.0251)	0.2136 (0.1131)
	Gemini 2.5 Pro	0.6138 (0.08)^b^	0.4037 (0.09)^b^	0.6846 (0.11)	0.4439 (0.11)
**Assessment scale**					
	GPT-4	0.63 (0.027)^c^	0.271 (0.05)^c^	0.797 (0.036)	0.233 (0.048)
	GPT-4o	0.681 (0.1587)^b^	0.213 (0.1587)^b^	0.9 (0.073)	0.223 (0.2389)
	GPT-o3	0.619 (0.06)^b^	0.283 (0.161)^b^	0.768 (0.09)	0.2682 (0.17)
	Claude 3.5	0.657 (0.109)^c^	0.33 (0.1153)^c^	0.7738 (0.1)	0.328 (0.1153)
	Gemini 2.5 Pro	0.518 (0.068)^b^	0.4822 (0.037)^b^	0.5524 (0.13)	0.478 (0.03)

^a^*P<*.001 for GPT-4 accuracy; *P<*.001 for GPT-4 *F*_1_-score; *P<*.001 for GPT-4o accuracy; *P<*.001 for GPT-4o *F*_1_-score; *P<*.001 for GPT-o3 accuracy; *P<*.001 for GPT-o3 *F*_1_-score (direct diagnosis versus code generation in non–assessment scale setups when marked in the “No assessment scale” section)

^b^No test conducted.

^c^*P=*.80, *P=*.60 for GPT-4 accuracy and *F*_1_-score; *P=*.30, *P*=.20 for Claude 3.5 accuracy and *F*_1_-score (assessment vs non-assessment).

Tables 3 and 4 [34] compare approaches on the AphasiaBank dataset against a baseline performance of 79% across metrics in the study by Cong et al [34]. All of our direct diagnosis conditions yielded a lower performance than this baseline. Our code generation conditions improved results significantly, with ChatGPT with GPT-o3 achieving the highest *F*_1_-score (0.865) and balanced specificity (0.92) and sensitivity (0.793), surpassing the baseline by Cong et al [34].

The results on the ASDBank dataset were compared against the baseline results from Chu et al [35], who achieved an *F*_1_-score of 0.85 and a high sensitivity of 0.94, although specificity was notably low at 0.2. Our direct diagnosis approaches struggled in comparison, with ChatGPT with GPT-4 and ChatGPT with GPT-o3 producing lower *F*_1_-scores (0.598 and 0.575, respectively) and poor specificity. The code generation condition significantly improved overall performance, with Claude 3.5 achieving the highest accuracy (0.68) and *F*_1_-score (0.6). The other models also showed improvement, but their performance on specificity and sensitivity was less consistent. Gemini 2.5 Pro was unable to provide ratings on the checklist due to content restrictions related to ethical guidelines.

For the DAIC-WOZ dataset, the studies by Dinkel et al [32] and Agrawal and Mishra [33] established strong baselines, achieving *F*_1_-scores of 0.84 and 0.91, respectively, along with high accuracy and sensitivity. In comparison, our direct diagnosis approaches showed inconsistent performance, with ChatGPT with GPT-4o and ChatGPT with GPT-4 achieving the highest accuracy (0.623) and *F*_1_-score (0.452)—notably low values—with even poorer results on the other metrics. While the code generation approaches yielded higher accuracy in some cases, they did not meaningfully improve overall performance as their *F*_1_-scores were significantly lower than those of the direct diagnosis condition.

We also note that most comparisons between assessment scale and no assessment scale conditions did not yield statistically significant differences except for ChatGPT with GPT-o3 and Gemini 2.5 Pro in the AphasiaBank direct diagnosis condition, which showed significant improvements in both accuracy and *F*_1_-score.

Overall, our findings reveal a substantial gap when using the 2 different approaches: code generation and direct diagnosis. While code generation and newer models seem to have improved performance compared to direct prompting, they still did not reach the levels reported in previous studies in most cases. Both approaches fell short of established benchmarks, underscoring the limitations of current LLM-based diagnostic methods that rely solely on prompting without model fine-tuning.

### Error Analysis

#### Overview

We first address the errors in the direct diagnosis approach, which did not appear to work well. We observed that most rounds of classification yielded close-to-random performances, especially for older models (ChatGPT with GPT-4 and ChatGPT with GPT-4o). Interestingly, we noticed patterns in the classification ratings produced, such as digits limited to only multiples of 3 or repeating sequences (eg, 3, 2, 1, 0, 3, 2, 1, 0). We present the percentage of rounds over 5 rounds of classification that followed such patterns in Table 9. This demonstrates that a direct diagnosis prompting strategy does not work well if models are presented with the entire dataset at once.

**Table 9:** Percentage of random predictions.

Database and approach			GPT-4 random predictions n=5 (%)		GPT-4o random predictions n=5 (%)		GPT-o3 random predictions n=5 (%)		Gemini 2.5 Pro random predictions (%)
**AphasiaBank**									
	Without assessment scale	80		60		0		0	
	With assessment scale	20		60		100		80	
**ASDBank**									
	Without assessment scale	20		100		0		80	
	With assessment scale	100		100		0		―^a^	
**DAIC-WOZ^b^ database**									
	Without assessment scale	40		80		0		20	
	With assessment scale	20		100		20		0	

^a^Not applicable.

^b^DAIC-WOZ: Distress Analysis Interview Corpus-Wizard-of-Oz.

For the code generation approach, we found some examples of text archetypes (ie, typical examples) that were frequently misclassified. These archetypes often reflect characteristics of the conditions. Common errors we observed are described in the following sections.

#### Repetitive Language and Filler Words (Aphasia)

The presence of repetitive language patterns and an increased frequency of filler words led to misclassification as a high proportion of false positives for aphasia. Control participants’ responses typically exhibited minimal repetition and filler word use. However, even a slight elevation in these linguistic elements frequently resulted in misclassification, with the chatbots erroneously classifying control participants as positives. Notably, misclassified false positives from almost all the chatbots contained these features.

#### Fragmented Sentences and Filler Words (ASD)

Transcripts containing filler words or fragmented sentences were misclassified in almost 100% of cases as false positives originating from individuals with ASD. With generative pretrained transformer models, this archetype was observed in most false-positive data points, indicating a consistent misclassification pattern. In contrast, Claude 3.5 exhibited a different trend because most misclassified points were false negatives. Claude 3.5 did not appear to excessively use the linguistic feature characteristic of this archetype.

#### Lack of Depressive Language (Depression)

Text lacking overt depressive indicators and conveying generally positive sentiments accounted for a large amount of false negatives. For instance, statements such as “uh I’d say maybe the fact that it’s a lot different than it was about ten years ago” and “I am pretty happy with the level of education I’ve gotten” often led to false negatives.

#### Excessive Amount of Laughter (Depression)

Texts containing instances of laughter were classified as false negatives in >70% of cases originating from control participants rather than individuals with depression.

#### Excessive Number of Sighs (Depression)

Texts containing references to sighing were categorized as false positives originating from individuals with depression. Over 30% of false-positive cases included this feature, indicating its disproportionate influence on the classification process.

#### Frequency of Occurrence of Archetypes

Table 10 details the frequency of occurrence of these archetypes. The observed misclassifications highlight the inherent constraints of relying on text-based methods for neurobehavioral diagnosis.

**Table 10:** Percentage of each text archetype in false-positive or false-negative data points in the code generation conditions averaged across folds.

Archetype and approach			GPT-4 (%)		GPT-4o (%)		GPT-o3 (%)		Claude 3.5 (%)		Gemini 2.5 Pro (%)
**Repetitive language and filler words (false positives)**											
	Without assessment scale	100		100		100		100		90	
	With assessment scale	100		100		0		100		85	
**Fragmented sentences and filler words (false positives)**											
	Without assessment scale	100		100		66.67		0		100	
	With assessment scale	100		100		66.67		0		―^a^	
**Lack of depressive language (false negatives)**											
	Without assessment scale	87.67		89.02		30		85		100	
	With assessment scale	87.67		89.09		100		79.09		100	
**Excessive amount of laughter (false negatives)**											
	Without assessment scale	88.36		88.34		78		90.7		96.77	
	With assessment scale	88.36		88.9		80.5		90.7		100	
**Excessive number of sighs (false positives)**											
	Without assessment scale	67.44		69.23		30.8		52		60.61	
	With assessment scale	65.12		74.19		29		52.17		100	

^a^Not available.

## Discussion

### Principal Findings

This study reveals the limitations of using LLMs for automated neurobehavioral classification. In both direct diagnosis conditions, we encountered significant limitations with these models, which tended to generate random or close-to-random predictions. The models occasionally refused to offer diagnoses, and when compelled to complete the tasks, the resulting classifications were not accurate. These challenges were even more pronounced with Claude 3.5 and Gemini 2.5 Pro, with which we faced difficulties generating any classification results or ratings in some conditions. The inclusion of assessment scales did not substantially improve performance as the ratings on scale items also appeared to be randomly assigned in most situations. Notably, in many of these conditions, we observed a concerning trend in which assessment scale ratings were often identical across participants regardless of individual differences in their text data.

It is important to note that previous studies have successfully achieved *F*_1_-scores of 70% to 80% using subsets of the ASDBank dataset and high performance (*F*_1_-scores of 80%-90%) using various methods on at least portions of the other 2 datasets [32-35]. In contrast, our results indicate that most direct diagnosis approaches and the code generated by these models were not able to attain similar results to those of previous studies. This discrepancy suggests a gap between the performance that ML models can potentially achieve and the outcomes observed in our study. This may be due to our relatively straightforward methodological approach.

Regarding the code generation condition, our findings suggest that LLM-generated ML pipelines show promising potential for improving diagnostic performance. Notably, on the AphasiaBank dataset, ChatGPT with GPT-o3 produced code that outperformed results reported in previous studies, although the choice of learning algorithms sometimes varied across conditions and lacked a clear rationale.

In the code generation condition using assessment scales, we observed that the code from the chatbots did not apply diagnostic thresholds as defined by the assessment scales but, instead, directly incorporated the ratings as ML features. The rating methods were simplistic, and the chatbots frequently implemented a keyword-counting algorithm to provide ratings for ASDBank and DAIC-WOZ. These ratings were then concatenated with features extracted from the feature extractor. This direct concatenation of features without sophisticated integration of diagnostic logic may explain why the assessment scale conditions did not lead to improved performance. More effective integration of these ratings in the generated code may help enhance future model performance.

We also observed that models with built-in chain-of-thought reasoning capabilities such as ChatGPT with GPT-o3 and Gemini 2.5 Pro exhibited improved performance under certain conditions. For instance, in the code generation tasks on the AphasiaBank dataset, these chain-of-thought models consistently outperformed others. Permutation tests conducted on the test sets across 5 cross-validation folds revealed statistically significant differences between models that used chain-of-thought reasoning and those that did not (ChatGPT with GPT-4 vs Gemini 2.5 Pro: accuracy *P=*.01, *F*_1_-score *P=*.03; ChatGPT with GPT-4 vs ChatGPT with GPT-o3: accuracy *P<*.001, *F*_1_-score *P<*.001; ChatGPT with GPT-4o vs Gemini 2.5 Pro: accuracy *P=*.01, *F*_1_-score *P=*.002; ChatGPT with GPT-4o vs ChatGPT with GPT-o3: accuracy *P<*.001, *F*_1_-score *P<*.001). While this improvement was not observed across all datasets (ie, DAIC-WOZ and ASDBank), the integration of structured prompting strategies appears to be a promising direction for future research.

In previous studies, human-in-the-loop processes have demonstrated promise for diagnostic classification tasks [39,40]. However, in such approaches, the human must remain more involved in the computational diagnosis procedure than simply prompting the LLM to generate a direct diagnosis, clinical rating, or classification code. In prior work for autism diagnostics, for example, humans have extracted the behavioral features—a task that requires the ability to interpret relatively subjective human behavior—leaving the ML models to perform the simpler task of the final classification given the human-derived features [41,42]. It is likely that humans performing at least some level of analysis of the data will need to continue to achieve clinically useful performance, and future prompt engineering approaches should explore these ideas more thoroughly.

### Limitations

We acknowledge several limitations of this study beyond the observed performance gaps.

First, the scope of our investigation was limited to 3 datasets, each representing a distinct neurobehavioral condition with relatively small sample sizes. This may constrain both the robustness and generalizability of our findings, as well as the models’ capacity to learn effectively.

Second, another limitation lies in the selection and applicability of the clinical checklists used in the assessment scale approach. In many cases, the patient transcripts lacked sufficient information to reliably rate all items on the scales, potentially resulting in random or invalid scores. Future work may consider using longer or more comprehensive patient transcripts or choosing assessment tools that are more tolerant of limited inputs.

Third, additional prompting strategies warrant exploration. While we observed performance gains from models that incorporated chain-of-thought reasoning by default, other prompting techniques may also enhance diagnostic accuracy.

Finally, all input data were presented to the models at once in a single file. This may have hindered their ability to process the content effectively. Presenting the data incrementally one instance at a time could reduce noise and improve prediction consistency.

### Conclusions

This study demonstrates that popular LLM-based chatbots remain inadequate for classifying neurobehavioral conditions from text transcripts even when prompted to incorporate clinical assessment scales into their evaluation strategy. We recommend that future research further investigate the limitations identified in this study and examine whether incorporating structured tools—such as assessment scales—can serve as a viable method to improve diagnostic accuracy for neurobehavioral conditions when using more sophisticated prompting strategies.

## Appendix

Multimedia Appendix 1 Prompts for large language models.

Multimedia Appendix 2 Code generated by GPT-o3 for ASDBank.

## Abbreviations

AI: artificial intelligence
ASD: autism spectrum disorder
DAIC-WOZ: Distress Analysis Interview Corpus-Wizard-of-Oz
LLM: large language model
ML: machine learning

## Data Availability

The datasets analyzed during this study are available in the AphasiaBank [27], ASDBank [26], and DAIC-WOZ [29] repositories.

## References

[1] Mathew A (2023). Is artificial intelligence a world changer? A case study of OpenAI’s Chat GPT. Recent Progr Sci Technol.

[2] Gao T, Yen H, Yu J, Chen D Enabling large language models to generate text with citations. ArXi.

[3] Biswas S (2023). Potential use of Chat GPT in global warming. Ann Biomed Eng.

[4] Lee YK, Lee I, Shin M, Bae S, Hahn S Chain of empathy: enhancing empathetic response of large language models based on psychotherapy models. ArXiv.

[5] Lai T, Shi Y, Du Z, Wu J, Fu K, Dou Y, Wang Z Psy-LLM: scaling up global mental health psychological services with ai-based large language models. ArXiv.

[6] Chen S, Zhang Z, Wu M, Zhu K (2023). Detection of multiple mental disorders from social media with two-stream psychiatric experts. Proceedings of the 2023 Conference on Empirical Methods in Natural Language Processing.

[7] Chen Z, Lu Y, Wang W (2023). Empowering psychotherapy with large language models: cognitive distortion detection through diagnosis of thought prompting. Proceedings of the Association for Computational Linguistics.

[8] Bhaumik R, Srivastava V, Jalali A, Ghosh S, Chandrasekharan R MindWatch: a smart cloud-based AI solution for suicide ideation detection leveraging large language models. medRxiv.

[9] Garg RK, Urs VL, Agarwal AA, Chaudhary SK, Paliwal V, Kar SK (2023). Exploring the role of ChatGPT in patient care (diagnosis and treatment) and medical research: a systematic review. Health Promot Perspect.

[10] Dergaa I, Fekih-Romdhane F, Hallit S, Loch AA, Glenn JM, Fessi MS, Ben Aissa M, Souissi N, Guelmami N, Swed S, El Omri A, Bragazzi NL, Ben Saad H (2023). ChatGPT is not ready yet for use in providing mental health assessment and interventions. Front Psychiatry.

[11] Clark LA, Cuthbert B, Lewis-Fernández R, Narrow WE, Reed GM (2017). Three approaches to understanding and classifying mental disorder: ICD-11, DSM-5, and the National Institute of Mental Health's Research Domain Criteria (RDoC). Psychol Sci Public Interest.

[12] (2013). Diagnostic and Statistical Manual of Mental Disorders, Fifth Edition.

[13] Rana M, Bhushan M (2022). Machine learning and deep learning approach for medical image analysis: diagnosis to detection. Multimed Tools Appl.

[14] Iyortsuun NK, Kim SH, Jhon M, Yang HJ, Pant S (2023). A review of machine learning and deep learning approaches on mental health diagnosis. Healthcare (Basel).

[15] Athaluri SA, Manthena SV, Kesapragada VS, Yarlagadda V, Dave T, Duddumpudi RT (2023). Exploring the boundaries of reality: investigating the phenomenon of artificial intelligence hallucination in scientific writing through ChatGPT references. Cureus.

[16] Ji Z, Lee N, Frieske R, Yu T, Su D, Xu Y, Ishii E, Bang YJ, Madotto A, Fung P (2023). Survey of hallucination in natural language generation. ACM Comput Surv.

[17] Ray PP (2023). ChatGPT: a comprehensive review on background, applications, key challenges, bias, ethics, limitations and future scope. Internet Things Cyber Phys Syst.

[18] Rasool A, Shahzad MI, Aslam H, Chan V Emotion-aware response generation using affect-enriched embeddings with LLMs. ArXiv.

[19] ChatGPT homepage. ChatGPT.

[20] Gemini Team Google Gemini: a family of highly capable multimodal models. ArXiv.

[21] Claude homepage. Claude.

[22] Amin MM, Cambria E, Schuller BW (2023). Will affective computing emerge from foundation models and general artificial intelligence? A first evaluation of ChatGPT. IEEE Intell Syst.

[23] Pandya A, Lodha P, Ganatra A (2024). Is ChatGPT ready to change mental healthcare? Challenges and considerations: a reality-check. Front Hum Dyn.

[24] Giadikiaroglou P, Lymperaiou M, Filandrianos G, Stamou G Puzzle solving using reasoning of large language models: a survey. ArXiv.

[25] Chen W, Ma X, Wang X, Cohen WW Program of thoughts prompting: disentangling computation from reasoning for numerical reasoning tasks. ArXiv.

[26] MacWhinney B (2019). Understanding spoken language through TalkBank. Behav Res Methods.

[27] Macwhinney B, Fromm D, Forbes M, Holland A (2011). AphasiaBank: methods for studying discourse. Aphasiology.

[28] TalkBank homepage. TalkBank.

[29] DeVault D, Georgila K, Artstein R, Morbini F, Traum D, Scherer S, Rizzo AS, Morency LP (2013). Verbal indicators of psychological distress in interactive dialogue with a virtual human. Proceedings of the SIGDIAL 2013 Conference.

[30] Bologna C What is aphasia?. National Aphasia Association.

[31] American Psychiatric Association (2022). Diagnostic and Statistical Manual of Mental Disorders, Fifth Edition, Text Revision (DSM-5-TR).

[32] Dinkel H, Wu M, Yu K Text-based depression detection on sparse data. ArXiv.

[33] Agrawal A, Mishra H (2024). Enhancing depression detection in clinical interviews: integration of fused BERT-BiLSTM Model and XGBoost. Proceedings of the 2024 10th International Conference on Computing and Artificial Intelligence.

[34] Cong Y, Lee J, LaCroix A (2024). Leveraging pre-trained large language models for aphasia detection in English and Chinese speakers. Proceedings of the 6th Clinical Natural Language Processing Workshop.

[35] Chu KC, Chiu YJ, Masud JH (2024). Applying machine learning to language problem analysis. Proceedings of the IEEE International Conference on Information Reuse and Integration for Data Science.

[36] Kertesz A (2007). Western Aphasia Battery–Revised.

[37] Baron-Cohen S, Wheelwright S, Skinner R, Martin J, Clubley E (2001). The autism-spectrum quotient (AQ): evidence from Asperger syndrome/high-functioning autism, males and females, scientists and mathematicians. J Autism Dev Disord.

[38] Burns DD (1999). The Feeling Good Handbook.

[39] Washington P (2024). A perspective on crowdsourcing and human-in-the-loop workflows in precision health. J Med Internet Res.

[40] Washington P, Wall DP (2023). A review of and roadmap for data science and machine learning for the neuropsychiatric phenotype of autism. Annu Rev Biomed Data Sci.

[41] Tariq Q, Daniels J, Schwartz JN, Washington P, Kalantarian H, Wall DP (2018). Mobile detection of autism through machine learning on home video: a development and prospective validation study. PLoS Med.

[42] Washington P, Tariq Q, Leblanc E, Chrisman B, Dunlap K, Kline A, Kalantarian H, Penev Y, Paskov K, Voss C, Stockham N, Varma M, Husic A, Kent J, Haber N, Winograd T, Wall DP (2021). Crowdsourced privacy-preserved feature tagging of short home videos for machine learning ASD detection. Sci Rep.

