# Supplementary material for: Aiding Large Language Models Using Clinical Scoresheets for Neurobehavioral Diagnostic Classification From Text: Algorithm Development and Validation
Source: JMIR AI. 2025 Oct 21;4:e75030. doi: 10.2196/75030 (PMC12587012; doi:10.2196/75030)
Supplement: Multimedia Appendix 1 [file ai_v4i1e75030_app1.docx]

## **Multimedia Appendix 1: prompts with LLMs**

**Direct Diagnosis (No Assessment Scale)**

Me: Here's a data point from one of the participants in the dataset. Could you take a look at it?

Chatbot: It looks like you've shared a dialogue between a user, Ellie, and an automated conversational agent. This conversation covers a range of topics including personal in-terests, career goals, and some insights into Ellie's family dynamics and social habits. Is there something specific you're analyzing or looking for in this conversation?

Me: I now have additional data points from 189 users. Could you help classify these patients as having depression or not?

Chatbot: To classify the patients into those with depression and those without, we'll need to process the text data you've provided, looking specifically for any explicit mentions of a depression diagnosis or symptoms strongly indicative of depression. From the snippets retrieved, here are examples of users being directly asked about depression and their responses:

1. Explicit confirmation of a depression diagnosis: "depression_diagnosed (have you been diagnosed with depression) yes".

2. Description of depression symptoms: "feeling hopeless all the time, not having enough energy to do things, feeling crummy about life".

Based on these and similar statements throughout the dataset, we can establish criteria for classification:

• Diagnosed with Depression: Any mention of being formally diagnosed by a pro-fessional.

• Symptoms Indicative of Depression: Descriptions of persistent sadness, loss of interest, fatigue, or other symptoms commonly associated with depression.

The next step would be to parse through each user's dialogue to flag these criteria. This can be automated with text analysis techniques to extract mentions of depression or its symptoms.

If you would like to proceed with an automated approach to classify these users based on the criteria, I can assist you in developing a script to process this text data. Let me know how you would like to proceed!

Me: Please choose an approach you regard as appropriate and provide a downloadable file.

Chatbot: I have processed the text data and classified the users based on mentions of depression or related symptoms. You can download the results from the following link:

(Continued with next iterations.)

**Direct Diagnosis (Assessment Scale)**

Me: Here's a data point from one of the participants in the dataset. Could you take a look at it?

Chatbot: Thanks for sharing this data! If you're looking to analyze or process this con-versation, could you let me know what specific aspects or tasks you're interested in ex-ploring? For instance, are you looking for sentiment analysis, thematic content extraction, or something else?

Me: Attached is Burn’s Depression Checklist along with the assessment scale to determine if a participant has depression:

(Include the assessment scale.)

Chatbot: Thanks for sharing the Burn's Depression Checklist and the scoring criteria. With this tool, you can assess individuals for various levels of depression based on their re-sponses to the 25-item questionnaire. Each item is rated on a scale from 0 to 4, indicating the severity or frequency of symptoms experienced over the past week.

If you're looking to apply this checklist to your dataset or need assistance in analyzing responses to determine the depression level of participants, let me know how I can assist further! Whether it's setting up a method to automate the scoring process or analyzing specific data entries, I'm here to help.

Me: I now have a dataset with 189 participants. Could you provide the ratings for these 25 items for each participant and list them in an output file?

Chatbot: The dataset you've uploaded contains text responses from participants and does not explicitly list scores for the 25 items of Burn's Depression Checklist. If you want to extract and score responses for each item, you will need to manually or programmatically match the responses to the checklist items based on sentiment analysis or keyword matching.

Would you like assistance with developing an approach to score these responses based on the content of the text data?

Me: I would like you to apply your pre-trained knowledge to rate 25 items for each participant. Please compile all the ratings into a file that I can download.

Chatbot: To accurately score the Burn’s Depression Checklist for each participant based on their responses, I would need to make inferences based on the emotional content and keywords within their responses. Since this involves a bit of interpretation, I will proceed with this approach:

1. Interpretation of Sentiments: I'll assess the sentiment in the responses related to each checklist item (e.g., feeling sad, discouraged, loss of interest) and assign scores ranging from 0 (Not At All) to 4 (Extremely).

2. Scoring: For each of the 25 items on the checklist, I'll infer a score based on the overall tone and content of the relevant sections of the conversation.

3. Output Preparation: The final scores for each participant will be compiled into a downloadable file.

Let me start by processing this data to generate the scores. This might take a moment.

I've generated the scores for the Burn’s Depression Checklist for each participant and saved them in a file. You can download the file from the following link:

(The chatbot generates a file and continues to the next iteration.)

**Code generation (No Assessment Scale)**

Me: Here is a dataset file that I would like to use for a machine learning project. Could you please read the dataset? We will use this dataset soon.

Chatbot: I've successfully read the data from the four files you provided. Here's a brief overview of the first few rows of each dataset: (removed)… Is there anything specific you would like to do with this data?

Me: Alright, I want to perform detection of aphasia patients in this data. Can you perform a stratified 5-fold cross-validation on your method and also report F1 scores, sensitivity, specificity and standard deviations?

Chatbot: To perform a stratified 5-fold cross-validation and report metrics like F1 scores, sensitivity, specificity, and their standard deviations, we first need to define a clearer criterion or chatbot to classify participants as having potential signs of aphasia or not. Since we are working with textual data, we can create a simple classification system based on the metrics we've computed—like repetition rate and average sentence length—or use more advanced NLP features.

However, let's first create a simplistic threshold-based classifier, which will classify a participant as showing potential signs of aphasia if their repetition rate exceeds a certain threshold and their average sentence length is below another threshold.

(The chatbot introduces the code. The chatbot created the y_true label and did not use the actual labels).

Me: I have the true labels, what you call “y_true”, in a variable called “all_label” as well as the corresponding input text data in a variable called “all_data”, so you don't need to generate y_true labels by yourself. Could you please use my “all_label” variable?

Chatbot: Great! Since you have actual labels (all_label) and the participants' text data (all_data), we can use these directly for the classification and cross-validation process. The approach remains mostly the same, but we'll now use your real data and labels.

Me: (If the results are not good:) The results are very bad, can you please try another method?

(The chatbot introduces its algorithm and provides the code.)

Chatbot: If the initial simplistic method based on repetition rate and average sentence length didn't provide satisfactory results, it's likely that we need a more sophisticated approach to capture the nuances of aphasia in text data. For such cases, using Natural Language Processing (NLP) techniques can be much more effective.

Me: There seems to be a bug: TypeError: sequence item 0: expected str instance, list found.

Chatbot: The error you're encountering occurs because the data structure wasn't as I assumed. It appears that all_data might still be a list of lists of lists, where each partici-pant's data is a list of sentences, and each sentence is a list of words. We need to correctly flatten this to a list of strings for TF-IDF vectorization.

(The chatbot provides new code.)

(Continue refining the algorithm until it is free of bugs and delivers performance above random chance)

**Code generation (Assessment Scale)**

Me: Here is a dataset file that I would like to use for a machine learning project. Could you please read in the dataset? We will use this dataset soon.

Chatbot: I've successfully read the data from the four files you provided. Here's a brief overview of the first few rows of each dataset: (removed)… Is there anything specific you would like to do with this data?

Me: Alright, please provide the details of your methodology for conducting a stratified 5-fold cross-validation procedure to predict the presence of Autism Spectrum Disorder (ASD) based on the given dataset.

Chatbot: To perform Stratified 5-fold cross-validation on your data to predict if patients have ASD, follow these steps using Python and scikit-learn:

1. Load and Preprocess Data: Ensure the data is loaded correctly and preprocessed to handle any missing values or categorical variables.

2. Define the Model: Set up a logistic regression model for binary classification.

3. Perform 5-Fold Cross-Validation: Use the cross_val_score function from scikit-learn to perform 5-fold cross-validation.

(The chatbot introduces the code)

Me: Would you please report F1 score, specificity and sensitivity, and their standard deviations?

Chatbot: To calculate F1 score, specificity, and sensitivity (recall) along with their standard deviations during Stratified 5-fold cross-validation, you can use the cross_val_score function for multiple metrics. Here's how you can modify the previous code to include these metrics:

(The chatbot introduces the code.)

Me: Now here's a list of the Autism-Spectrum Quotient (AQ) scale items. We aim to in-corporate these ratings into the classification model. According to the scale, users with a score exceeding 26 are considered to have ASD.

Chatbot: To incorporate the Autism Quotient (AQ) ratings into the code and calculate scores, we need to implement the scoring rules you provided. We'll integrate these scores into our data preprocessing step, and then use these scores as features in the model.

Here’s how to do it:

1. Define the scoring rules: Create a function to calculate AQ scores based on the re-sponses.

2. Include AQ scores in the dataset: Use these scores as additional features for the model.

3. Update the pipeline to include these features.

(The chatbot introduces the code.)

Ensure to replace the placeholder paths with the actual paths to your data files and adjust any specific details according to your dataset structure. This should now incorporate AQ scores into the model training and evaluation process.

Me: The text data provided does not include a pre-generated aq_responses variable. In-stead, we will need to programmatically derive the Autism-Spectrum Quotient (AQ) scale responses from the textual information available in the dataset.

Chatbot: Got it. We'll need to create a synthetic dataset for AQ responses to integrate with the main dataset. Here's the updated code to include the generation of AQ responses and their integration into the dataset:

1. Generate AQ responses: Create synthetic AQ responses for demonstration.

2. Calculate AQ scores: Use the provided AQ scoring rules.

3. Integrate AQ scores with text data: Use these scores as additional features for the model.

(The chatbot introduces the code.)

Me: Please refrain from generating synthetic ratings and instead focus on producing ratings derived directly from the provided text data.

Chatbot: I understand now. Here's a step-by-step approach to generate the responses based on the provided text data, calculate the AQ scores, identify participants with autism, and perform the stratified 5-fold cross-validation. Step-by-Step Implementation:

1. Extract Responses from text Data: Process the text data to extract responses.

2. Calculate AQ Scores: Calculate the AQ score for each participant based on their re-sponses.

3. Identify Participants with Autism: Determine which participants have an AQ score greater than 26.

4. Prepare Data for Cross-Validation: Use logistic regression to classify the data.

5. Perform Stratified 5-Fold Cross-Validation: Report the F1 score, specificity, sensitivity, and their standard deviations.

(The chatbot introduces the code.)

Me: The results indicate that the F1 scores and sensitivities are currently at zero. Could you please refine your methodology to enhance performance outcomes?

Chatbot: To avoid having a lot of zeros in the F1 scores and sensitivity, we can consider the following strategies:

1. Balance the Classes: Ensure that the dataset is balanced so that the model has enough samples of each class to learn from. This can be done through oversampling the minority class or undersampling the majority class.

2. Feature Engineering: Improve feature representation to make the data more in-formative for the model.

3. Model Tuning: Adjust hyperparameters of the model to better handle imbalanced data.

Here's an updated version of the code that includes class balancing using the SMOTE technique for oversampling the minority class:

(The chatbot introduces the code.)

(Continue refining the algorithm until it is free of bugs and delivers performance above random chance.)
