# Supplementary material for: Aiding Large Language Models Using Clinical Scoresheets for Neurobehavioral Diagnostic Classification From Text: Algorithm Development and Validation
Source: JMIR AI. 2025 Oct 21;4:e75030. doi: 10.2196/75030 (PMC12587012; doi:10.2196/75030)
Supplement: Multimedia Appendix 2 [file ai_v4i1e75030_app2.docx]

**Multimedia Appendix 2: Code generated by the GPT-o3, ASDBank**

import ast

from pathlib import Path

import numpy as np

from sklearn.feature_extraction.text import TfidfVectorizer

from sklearn.linear_model import LogisticRegression

from sklearn.metrics import classification_report, accuracy_score, f1_score

from sklearn.model_selection import StratifiedKFold, cross_validate

# ---- 1. Load data -----------------------------------------------------------

def load_data(data_path: str, label_path: str):

raw_text = Path(data_path).read_text(encoding="utf-8").strip()

# Auto-detect storage format ------------------------------------------------

if raw_text.startswith("[") and raw_text.endswith("]"):

# File is one big literal list -> ast.literal_eval

examples = ast.literal_eval(raw_text)

else:

# One example per line -> literal-eval each line

examples = [ast.literal_eval(line) for line in raw_text.splitlines() if line]

# If examples are token lists, join them into strings so TF-IDF sees words

if examples and isinstance(examples[0], (list, tuple)):

examples = [" ".join(tokens) for tokens in examples]

labels = [ast.literal_eval(line.strip()) for line in Path(label_path).read_text(encoding="utf-8").splitlines() if line]

assert len(examples) == len(labels), "Data / label size mismatch!"

return examples, np.array(labels)

X, y = all_data_asd, all_label_asd

y = np.asarray(y) # guarantee ndarray

# ---- 2. Build a text-classification pipeline --------------------------------

tfidf = TfidfVectorizer(

lowercase=True,

max_df=0.9,

min_df=2,

ngram_range=(1, 2), # unigrams + bigrams

sublinear_tf=True,

)

clf = LogisticRegression(

penalty="l2",

C=1.0,

solver="liblinear",

max_iter=1000,

class_weight="balanced",

n_jobs=-1,

)

# ---- 3. Stratified 5-fold cross-validation ----------------------------------

from sklearn.metrics import (

make_scorer,

recall_score,

f1_score, # only needed if you customise F1 further

)

cv = StratifiedKFold(n_splits=5, shuffle=True, random_state=42)

# -------------------------------------------------

# 1. Define the scorers

# -------------------------------------------------

SCORING = {

# built-in identifiers

"accuracy": "accuracy",

"f1_micro": "f1_micro", # overall F1 weighted by support

# custom callables

"sensitivity": make_scorer(recall_score, pos_label=1), # True-Positive Rate

"specificity": make_scorer(recall_score, pos_label=0), # True-Negative Rate

}

# -------------------------------------------------

# 2. Run stratified 5-fold CV

# -------------------------------------------------

results = cross_validate(

estimator=clf,

X=tfidf.fit_transform(X), # TF-IDF already defined/fitted earlier

y=y,

cv=cv, # StratifiedKFold(5, shuffle=True, random_state=42)

scoring=SCORING,

return_train_score=False,

n_jobs=-1,

)

# -------------------------------------------------

# 3. Aggregate & display

# -------------------------------------------------

print("Stratified 5-fold results (mean ± SD)")

for metric in SCORING:

fold_scores = results[f"test_{metric}"]

print(f"{metric:12s}: {fold_scores.mean():.3f} ± {fold_scores.std():.3f}")

# ---- 5. Optional: full classification report for each fold ------------------

print("\nDetailed per-fold reports")

for i, (train_idx, test_idx) in enumerate(cv.split(X, y), 1):

X_train, X_test = np.asarray(X)[train_idx], np.asarray(X)[test_idx]

y_train, y_test = y[train_idx], y[test_idx]

X_train_vec = tfidf.transform(X_train) # vectorizer already fitted above

X_test_vec = tfidf.transform(X_test)

clf.fit(X_train_vec, y_train)

y_pred = clf.predict(X_test_vec)

print(f"\nFold {i}")

print(classification_report(y_test, y_pred, digits=3))
